# Kinetic fractionation of Mg isotopes during chemical diffusion in aqueous solutions: A reappraisal

**DOI:** 10.1016/j.fmre.2024.11.010

**Published:** 2024-12-25

**Authors:** Weiqiang Li, Xianglong Luo, Zhihan Ji, Chuan Liu

**Affiliations:** a State Key Laboratory for Mineral Deposits Research, School of Earth Sciences and Engineering, Nanjing University, Nanjing 210023, China; b Frontiers Science Center for Critical Earth Material Cycling, Nanjing University, Nanjing 210023, China

**Keywords:** Chemical diffusion, Mg isotope, Fractionation, Aqueous Mg, Temperature effect

## Abstract

Diffusion is a basic process that could result in isotopic variability in nature, and diffusion-driven Mg isotope fractionation has been widely reported in a variety of magmatic processes. Whether chemical diffusion of Mg aquo ions in aqueous solution is associated with significant isotope fractionation, however, had been contentious. Addressing this issue can advance our understanding of the molecular configuration and movement of cations in aqueous solutions. Here, we modified the recently reported “diffusion cell” method to investigate the isotopic effect of diffusion of aqueous Mg under various conditions, with emphasis on the effects of temperature and anions. The experimental results show that in aqueous solutions, lighter Mg isotopes diffuse faster than the heavier isotopes. The ratio of diffusion coefficients between ^26^Mg and ^24^Mg (D_26Mg_/D_24Mg_, or kinetic isotope fractionation factor α) ranges from 0.99990 ± 0.00003 (1σ) to 0.99982 ± 0.00003 (1σ), depending on the specific experimental conditions. Notably, the magnitude of diffusion-induced Mg isotope fractionation in aqueous solution increases with higher temperature, and is greater for MgCl_2_ and Mg(NO_3_)_2_ than for MgSO_4_. Based on these new findings and a comparison with published data on diffusion-driven isotope fractionation for other divalent elements, we suggest that the mass dependency of isotope fractionation during chemical diffusion in aqueous solutions is regulated by the strength of ion-water interactions. Stronger ion-water interactions result in more pronounced hydrodynamic behavior at a molecular level for the diffusing ions, leading to less significant isotope fractionation in aqueous fluids.

## Graphical abstract

**Figure:**
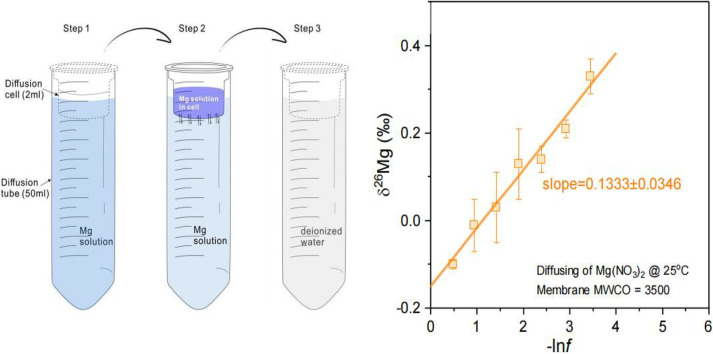

## 1 Introduction

Diffusion is a ubiquitous kinetic process in nature. For example, growth or dissolution of minerals in melts or aqueous solutions, redistribution of elements or isotopes in heterogeneous rocks or minerals, and inter- and intra-cellular dispersion of ions and molecules within organisms, are associated with diffusion. Diffusion of elements is commonly accompanied by isotope fractionation, because the mass of the diffusing particle is a basic factor that controls diffusion coefficient [[1], [2], [3]]. Isotopic fractionation during diffusion in gaseous, aqueous, and solid phases could contribute to the stable isotope variability of different elements in terrestrial and extraterrestrial samples [1]. Therefore, mechanistic and quantitative understandings of isotope fractionation behavior during diffusion are important for interpreting stable isotope signatures in natural samples that can be used to constrain the relevant geological, cosmochemical, and biological processes.

Magnesium is a major constituent of the Earth’s crust and mantle and is also the second most abundant cation in seawater and an essential micro-nutrient in organisms. Magnesium has three stable isotopes and Mg isotopic compositions vary remarkably in terrestrial and extraterrestrial samples [4], and a substantial proportion of the Mg isotope variability in igneous rocks is related to kinetic processes associated with diffusion. Large (sometimes extreme) Mg isotope fractionation had been reported from mineral grains of olivine and ilmenite in mafic magmatic systems [[5], [6], [7], [8], [9]] and at the contact boundary between mafic and felsic igneous rocks [[10], [11], [12]]. Such signatures were interpreted to be produced by elemental diffusion between minerals and melts under chemical disequilibrium. Efforts had also been undertaken to quantitatively constrain the isotopic behavior of Mg during various high-temperature diffusional processes by laboratory experiments [10,[13], [14], [15]] and theoretical calculations [16].

In comparison, there had been fewer studies on Mg isotope fractionation during diffusion in aqueous solution. One early experimental investigation by Richter et al. yielded a Mg isotope fractionation factor (α, α = D_26Mg_/D_24Mg_) of 1.00006 ± 0.00012 [3]. If this factor is correct, it would mean that the heavy Mg isotopes (^26^Mg) diffuse slightly faster than the light Mg isotopes (^24^Mg) in aqueous solutions, a case that violates the classic diffusion theories [1]. Alternatively, the reported factor can be interpreted to be equal to unity within analytical uncertainty, meaning that there is essentially no kinetic Mg isotope fractionation during diffusion of Mg^2+^ ions in aqueous solution. This is still intriguing considering the large relative mass difference (8%) between ^26^Mg and ^24^Mg that would warrant significant isotope fractionation according to simple kinetic transport theories. Bourg and Sposito [17] used molecular dynamics simulations to calculate the kinetic Mg isotope fractionation during self-diffusion of Mg^2+^ and confirmed that D_26Mg_/D_24Mg_ is close to unity, but the uncertainty associated with the calculation was even greater than the experimental study of Richter et al. [3]. Moreover, the study of Bourg and Sposito [17] was based on calculations of self-diffusion coefficient of cations, which is a simplification of natural processes because speciation and diffusion of cations in aqueous solutions could be affected by counter ions (anions).

Recently, Li et al. [18] experimentally obtained an α factor of = 0.999877 ± 0.000010 for Mg isotopes, which is distinctly lower (i.e., outside the 2σ error envelopes) than that of Richter et al. [3]. The two studies had some notable differences in experimental settings, such that Li et al. [18] used Mg(NO_3_)_2_ whereas Richter et al. [3] used MgCl_2_ as the solute, and in experiments of Li et al. [18] the solute diffused through a membrane whereas there was no membrane in the experiments of Richter et al. [3]. The potential effects of interaction between aqueous ions and membrane, and the effects of aqueous speciation in cations in aqueous solutions were not explored in the study of Li et al. [18]. Further, neither experiments of Li et al. [18] nor Richter et al. [3] evaluated the effects of temperature. Ascertaining these effects is crucial for understanding the kinetic isotope fractionation of cations during chemical diffusion in aqueous solutions, as well as the molecular-level behavior of cations. In this study, we revisited the problem of isotope fractionation of Mg during its chemical diffusion in aqueous solutions using a modified version of the diffusion cell method that was reported by Li et al. [18]. We systematically investigated how temperature and anions affect the elemental and isotopic behaviors of Mg during diffusion in aqueous solutions. We report that chemical diffusion of aqueous Mg ion is associated with a measurable Mg isotope fractionation (α = 0.99990 ± 0.00003 to 0.99982 ± 0.00003) and the kinetic Mg isotope fractionation factors show dependence on experimental conditions including temperature and the type of anions. The implications of the diffusion-induced Mg isotope fractionation on molecular structure of Mg ions in aqueous solutions and interpretations on Mg isotope data from low temperature systems are discussed.

## 2 Experimental design

There are two different approaches to experimentally determine diffusion-driven kinetic isotope fractionation factors. One approach is to sample a diffusion profile, and use the spatial variation of isotopic ratios to derive fractionation factor [19,20]; the other approach is to measure the temporal changes of the isotope ratios of one or more reservoirs that undergo diffusion [3,21,22,23]. Essentially the experimental methods of Richter et al. [3] and Li et al. [18] belong to the latter.

**Fig. 1:**
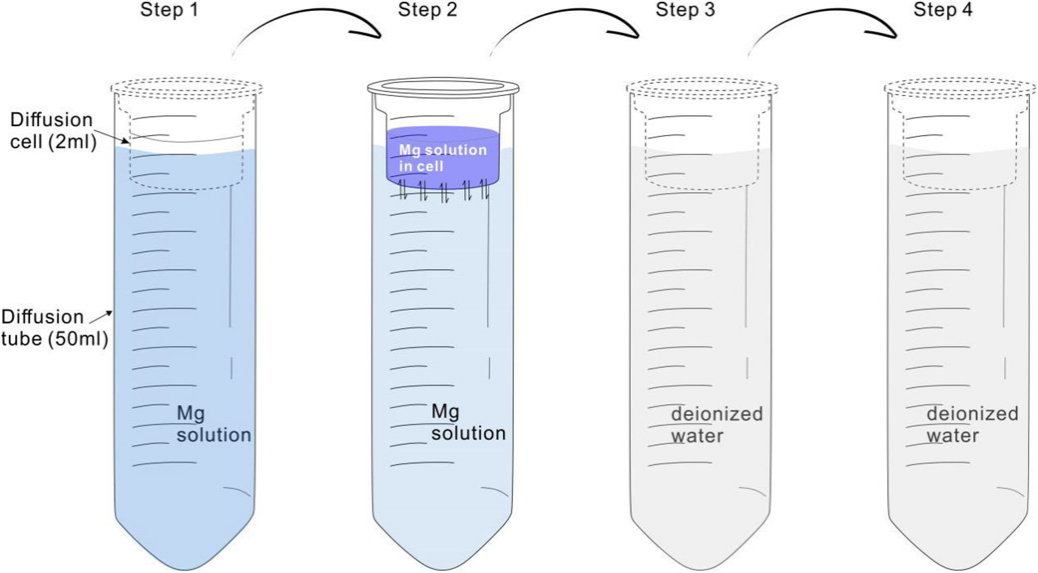
**Sketch showing the experimental set up of this study**.

In this study, we further modified the “diffusion cell” method of Li et al. [18] to investigate Mg isotope fractionation during diffusion in aqueous solution. The experimental setup is illustrated in Fig. 1. For a typical experiment, a Mg solution was added into a diffusion cell that had a flat dialysis membrane at the bottom, and the diffusion cell was then placed in a test tube that contained a significantly larger volume of Mg-free fluid (i.e., deionized water) to allow diffusional loss of Mg into the tube. After a certain length of time, the diffusion cell was placed into a new test tube that contained fresh Mg-free fluid for diffusion, and such process was repeated. After the diffusion experiment, the solutions remaining in the diffusion cell and the test tube were analyzed for elemental concentration and isotopic composition.

The mathematical method to derive α is the same as that of Li et al. [18] and is introduced as below. According to Fick’s first law, the diffusional flux (J) across the membrane follows:

(1) $$J=-DK\left(C_{cell}-C_{tube}\right)$$

where *D* is the diffusion coefficient of the dissolved component of interest, *C* is the concentration of the component of interest in the diffusion cell or in the tube, and K is a constant related to the property of the membrane including the thickness (L), cross section area *A*, and effective porosity of the membrane (Φ), as K *=* *AΦ/*L. Because the volume of tube is far greater than the cell (i.e., *V_tube_/V_cell_* = 25 in our study), and the diffusion cell is moved to a new tube frequently during experiment, Mg concentration in the tube is much lower than that in the cell (*C_tube_ << C_cell_*), thus the flux J can be considered proportional to C_cell_ (i.e., J *= -DKC_cell_*) by approximation. This is a Rayleigh distillation relation and a time integration leads to

(2) $$C_{cell}^{i}/C_{cell}^{0}=e^{-DKt}=f$$

where $C_{Cell}^{0}$ is the initial concentration of a component of interest (Mg in this study) and $C_{Cell}^{i}$ is the concentration after diffusion by a period of *t*. Eq. 2 defines a key parameter *f* as the fraction of solute remaining in the diffusion cell after it has been immersed in *i* tubes.

Assuming that the diffusion experiment has been conducted in *n* tubes (as shown in Fig. 1), then based on elemental mass balance, we have an alternative expression of *f*, which is

(3) $$f=\frac{\sum_{i+1}^{n}{(C}_{\text{tube}}^{i+1}\times V_{\text{tube}})+C_{\text{cell}}^{n}\times V_{\text{cell}}}{C_{\text{cell}}^{0}\times V_{\text{cell}}}=\frac{\sum_{i+1}^{n}{(C}_{\text{tube}}^{i+1}\times V_{\text{tube}})+C_{\text{cell}}^{n}\times V_{\text{cell}}}{\sum_{1}^{n}{(C}_{\text{tube}}^{i}\times V_{\text{tube}})+C_{\text{cell}}^{n}\times V_{\text{cell}}}$$

where V is the volume of aqueous solution in the tube or cell.

Eq. 3 is important because for the diffusion experiment in this study, *f* at any stage can be calculated based on Mg concentration analysis of the solutions in all diffusion tubes and the final diffusion cell. This avoids sampling of fluids in the diffusion cell during the experiment and allows conduction of the diffusion experiment by automation.

Now we consider diffusion of two isotopes (*l* and h) in the system, which have diffusion coefficient of *D* and D*, respectively. The evolution of isotopic composition (R) within the cell can be expressed as

(4) $$\frac{R_{cell}^{i}}{R_{cell}^{0}}=\frac{C_{cell}^{h,i}/C_{cell}^{l,i}}{C_{cell}^{h,0}/C_{cell}^{l,0}}=\left(\frac{C_{cell}^{h,i}}{C_{cell}^{h,0}}\right)/\left(\frac{C_{cell}^{l,i}}{C_{cell}^{l,0}}\right)$$

Based on the above equations, we can follow the mathematical techniques in ref [3] to derive that the isotope composition of solute in the diffusion tube (δ_tube_) is linearly correlated with ln*f*:

(5) $$\delta_{tube}^{i}=\ln f\cdot \left(D^{*}/D-1\right)\cdot 1000+\left(\delta_{cell}^{0}+k\right)$$

where *k* is a constant related to the membrane of the diffusion cell and the diffusing characteristics of the solute. Based on Eq. 5, the kinetic isotope fractionation factor associated with diffusion (D*/D, or α) can be derived by systematic analysis of isotopic composition of the aqueous solutions in the tubes in a diffusion experiment as illustrated in Fig. 1. Specifically, Eq. 5 shows that in a plot of *δ* versus ln*f*, the values for δ_tube_ follow a linear trend with a slope of (D*/D-1)∙1000. Details of the derivation of Eq. 5 from (1), (2), (3), (4) are given in Appendix A1; additional related mathematical proofs are given in the appendix of Li et al. [18].

## 3 Experiments and methods

### 3.1 Diffusion experiments

A commercial Slide-A-Lyer® dialysis kit from Thermo Scientific for life sciences was used as the diffusion cell, the diffusion cell is cylindrical in shape and has a dialysis membrane at the bottom. The membranes of the Slide-A-Lyer ® kits have two different pore sizes, which correspond to molecular weight cut off (MWCO) of 3500 and 20,000 Daltons, respectively. Prior to experiments, the diffusion cell was cleaned by soaking in 2% HNO_3_ overnight, then rinsed by deionized water multiple times. The blanks of Mg and Ca from the membrane of the diffusion cell were 83–75 and 460–520 ng, respectively.

For a typical diffusion experiment, 2 ml Mg solution was transferred to the diffusion cell. The diffusion cell was placed into one of the 50 ml centrifuge tubes in a rack immersed in the water bath. The temperature of the water bath was preset at 5, 25, 50, or 70 °C within ±0.1 °C. To ensure exact time of diffusion for each tube, a programmed robotic arm was used to move the diffusion cell from one centrifuge tube to the next (Appendix A2). The duration that the diffusion cell stayed in each tube was set based on prior trials so that 30% to 50% of Mg diffused out of the cell for each tube.

Four sets of diffusion experiments were performed. For all experiments, the initial Mg solutions were prepared by dissolving analytical grade MgCl_2_, Mg(NO_3_)_2_, or MgSO_4_ in deionized water to 0.4 M or 0.5 M, thus the initial solution had a typical Mg concentration of ca. 10,000 or ca. 12,000 ppm. Experiment set 1 assessed the effect of membrane pore size. In such experiments, the temperature was kept at 25 °C and 2 ml MgCl_2_ solution was added to the diffusion cells that had two different membranes (MWCO = 3500 and 20,000). Experiment set 2 assessed the effect of temperature, so diffusion experiments for MgCl_2_ in a diffusion cell with a membrane of 3500 Daltons MWCO were performed at 5, 25, 50, and 70 °C. Experiment set 3 assessed the effect of anions on the diffusion of Mg. In such experiments, the temperature was kept at 25 °C and the diffusion cell had a membrane with MWCO of 3500 Daltons, but 2 ml MgCl_2_, Mg(NO_3_)_2_, and MgSO_4_ solutions were added to the diffusion cell in different experiments. Experiment set 4 assessed the effect of background electrolyte on the diffusion of Mg. For this experiment, 2 ml 0.4 M MgCl_2_ in the diffusion cell with a membrane of 3500 Daltons was diffused into 0.8 M HCl solutions at 25 °C, in contrast to other diffusion experiments where the Mg solutions were diffused into pure water. During the experiments, two batches of different MgCl_2_ salts were used, which have different starting Mg isotope compositions. The diffusion experiments generally stopped after *f* was less than 0.005 (i.e., Mg content of aqueous solution remaining in the diffusion cell was below 60 ppm). Then the solutions in the cells and tubes were used for elemental and Mg isotope analysis.

In addition to the diffusion experiments, a batch of adsorption-desorption experiments were performed to assess the isotopic effect on the membrane. A pre-cleaned membrane of 3500 Daltons MWCO was immersed in a 40 ppm solution of MgCl_2_, Mg(NO_3_)_2_, or MgSO_4_ overnight for equilibration, and then it was rinsed with deionized water twice to remove surface Mg-bearing fluids. After that the membrane was soaked in 7 mL 2% HNO_3_ overnight to release the Mg adsorbed on the membrane. The Mg solutions were then measured for concentration and isotopic ratios.

### 3.2 Chemical and isotopic analyses

Element concentration analysis was performed on a Skyray type ICP3000 inductively coupled plasma optical emission spectrometer (ICP-OES). A series of gravimetrically prepared multi-element standard solutions were used to generate the working curve of concentration calculation. The accuracy of elemental measurement was better than ±5%.

For experiments of MgSO_4_, an aliquot of the MgSO_4_ solution that contained 50–100 μg of Mg was dried in a teflon beaker, then dissolved in 1 mL 0.2 M HCl, and processed on a cation exchange column to remove sulfate, following the method of [24]. For experiments of MgCl_2_ and Mg(NO_3_)_2_, the Mg solutions were dried in teflon beakers on a hot plate, then redissolved in 0.2 mL concentrated HNO_3_ and evaporated to dryness. These samples were not subjected to column chemistry because of the high purity of the Mg solutions before and after the diffusion experiments. The treated Mg samples were diluted to 0.50 ± 0.05 ppm in 2% HNO_3_ and analyzed for Mg isotope ratios.

Magnesium isotope analysis was performed on a Nu Plasma 1700 Sapphire multi-collector inductively-coupled-plasma mass spectrometer (MC-ICP-MS) at the State Key Laboratory for Mineral Deposit Research, School of Earth Sciences and Engineering, Nanjing University. The instrument is equipped with a dual-pass collision cell system, which enables analysis with or without collision cell. In this study, the instrument was operating on a high energy path without collision cell, which had a behavior equivalent to a standard Nu Plasma 1700 MC-ICP-MS. The mass spectrometry procedure has been reported in a recent study [25]. Briefly, Mg solutions were introduced into a hot ICP (forward power = 1300 w) by a Glass Expansion concentric nebulizer at a flow rate of 100 μL/min, through a cyclonic spray chamber Peltier-cooled to 7 °C. The sample-standard-sample bracketing method was used for correction of mass bias and instrument drift. Each analysis consisted of 100 s of washout time, and 200 s (4 s × 40) of integration on Mg signals. 10^11^ Ω resistors were used for all Faraday cups that collected the three Mg isotopes, and a 0.5 ppm Mg solution yielded 8–12 Vs of total signals. Internal precision of ^26/24^Mg ratio in a single isotopic analysis was better than ± 0.04‰, and the external reproducibility of Mg isotope analysis was better than ± 0.10‰ for ^26/24^Mg ratios based on repeated analysis of multiple Mg isotope standards.

### 3.3 Aqueous mg speciation calculation

Speciation of Mg in the aqueous solutions and the relative proportions of these Mg species were calculated using PHREEQC Interactive 3.5.0 [26]. The calculations were performed using the *llnl.dat* database, which incorporates the thermodynamic constants for Mg^2+^hydrolysis [27]. Speciation calculations were performed for solutions with Mg concentration that varied over the range of the experimental conditions (i.e., 0.5 M Mg to 0.0001 M Mg) at different temperatures (i.e., 5, 25, 50, 70 °C).

## 4 Results

### 4.1 Elemental results for the diffusion experiments into water

**Fig. 2:**
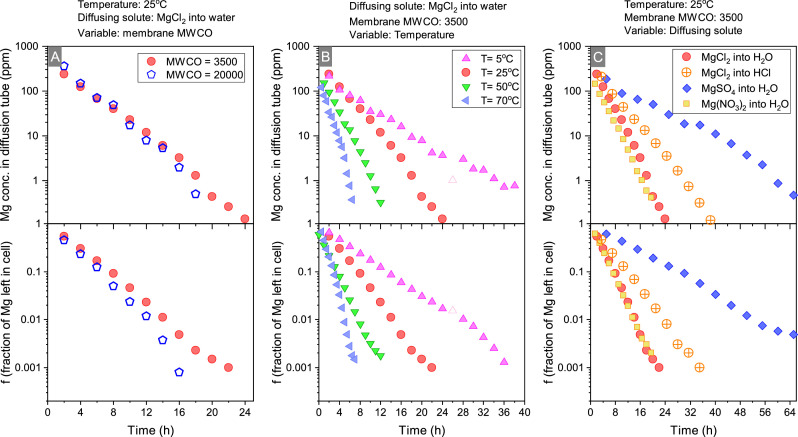
**Summary of the change of Mg concentration in diffusion tube and the corresponding *f* (fraction of Mg remaining in diffusion cell) with time for different sets of experiments**. In panel A, the diffusion experiments were performed at 25 °C, with MgCl_2_ solutions diffusing into water, but through two different membranes. In panel B, the diffusion experiments were conducted by diffusing MgCl_2_ into water through the same membrane, but at four different temperatures. In panel C, the diffusion experiments were conducted at the same temperature using the same membrane, but the dissolved Mg salts were different.

The results of elemental analysis are summarized in Fig. 2 and Appendix A3. In all experiments, the Mg concentration in the diffusion tube decreased exponentially with time (Fig. 2). There is only one outlier data in Fig. 2B (shown as unfilled triangle) for the MgCl_2_ diffusion experiment at 5 °C, which was likely due to analytical error. The fraction of Mg remaining in the diffusion cell (*f*) after each time point was calculated following Eq. 3 and plotted in Fig. 2. The excellent linearity (*R*^2^ > 0.95 for all experiments) of the data trends indicates that diffusion of Mg ions from the cell to the tube followed a Rayleigh behavior, and the diffusion coefficient (D) remained consistent throughout each of the different diffusion experiments.

The elemental results suggest that the pore size of membrane has barely resolvable effect on the diffusion of aqueous Mg (Fig. 2A). Given the same temperature (25 °C) and solute (MgCl_2_), the time to lose 99% of MgCl_2_ (*f* = 0.01) was about 14 and 12 h for diffusion cell that had membrane MWCO of 3500 and 20,000, respectively. Because the membrane’s MWCO is controlled by pore size, the results show that larger pore size facilitates faster diffusion, but the difference is insignificant relative to other factors. By contrast, temperature has a profound influence on the speed of diffusion (Fig. 2B). For a diffusion cell with a membrane MWCO of 3500, it took about 4 h to lose 99% (i.e., *f* = 0.01) of MgCl_2_ in the diffusion cell at 70 °C, whereas the time to achieve *f* of 0.01 increased to about 14 h at 25 °C and about 28 h at 5 °C. Diffusion of Mg^2+^ is also strongly influenced by the anion. Given the same diffusion cell (membrane MWCO = 3500) and temperature (25 °C), Mg(NO_3_)_2_ diffuses almost as fast as MgCl_2_, whereas MgSO_4_ diffuses much slower than MgCl_2_ (Fig. 2C).

### 4.2 Mg isotope results for the diffusion experiments into water

**Fig. 3:**
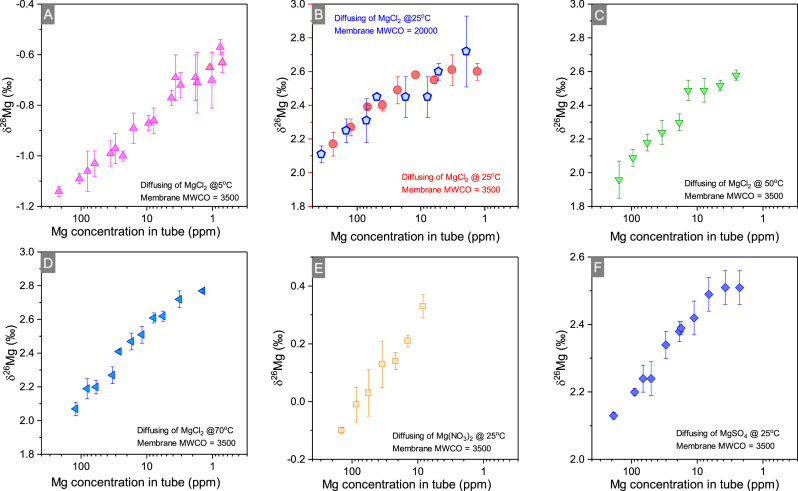
**Plots of δ^26^Mg versus Mg concentration of the Mg solutions in the diffusion tube for different experiments**. (A) Diffusion of MgCl_2_ solution at 5 °C through a membrane with MWCO of 3500 Dalton; (B) Comparison of results of diffusion of MgCl_2_ solution at 25 °C through membrane with MWCO of 3500 and 20,000 Dalton; (C) Diffusion of MgCl_2_ solution at 50 °C through a membrane with MWCO of 3500 Dalton; (D) Diffusion of MgCl_2_ solution at 70 °C through a membrane with MWCO of 3500 Dalton; (E) Diffusion of Mg(NO_3_)_2_ solution at 25 °C through a membrane with MWCO of 3500 Dalton; (F) Diffusion of MgSO_4_ solution at 25 °C through a membrane with MWCO of 3500 Dalton.

The Mg isotope composition of Mg solutions in the tube after the diffusion experiments is plotted against Mg concentration (in log_10_ scale) in Fig. 3 for different experiments (data are tabulated in Appendix A3). There are several striking features in the data plots. First, in all diffusion experiment there is a distinct trend of increasing δ^26^Mg values of the Mg solution with decreasing Mg concentration, regardless of experimental conditions (i.e., temperature, solute, membrane). Second, the magnitude of increase in δ^26^Mg of the Mg solution in the tubes are broadly similar among different experiments, which are around 0.6‰ ± 0.2‰. Third, the data plots are generally linear in Fig. 3; however, the slope of the data trend flattened (smaller or no increase in δ^26^Mg with decreasing Mg concentration) when the Mg content in the tube was below 3–5 ppm. The results clearly show that diffusion of Mg ions is accompanied with resolvable Mg isotope fractionation.

### 4.3 Elemental and isotopic results for MgCl_2_ diffusion into HCl

In addition to experiments of diffusing Mg salt solutions into pure water, we performed an experiment of diffusing a 0.4 M MgCl_2_ solution into 0.8 M HCl, to evaluate the effect of background electrolyte. The elemental and isotopic results are tabulated in Appendix A3, which show that the Mg in the diffusion tubes decreased following an exponential trend, similar to those of Mg solutions diffusing into water. However, it is remarkable that the rate of MgCl_2_ diffusing into HCl was significantly slower than that of MgCl_2_ diffusing into H_2_O under the same conditions (same membrane and temperature). It took about 24 h to lose 99% of MgCl_2_ (*f* = 0.01) by diffusing into 0.8 M HCl, compared to 14 h in diffusion experiments into water through the same membrane (MWCO = 3500) at 25 °C. The Mg isotope results of this experiment are also highly similar to those of the experiment of MgCl_2_ diffusion into water, as the δ^26^Mg values of Mg in diffusion tubes increased by 0.6 ‰ when the Mg content decreased to 1.6 ppm.

### 4.4 Elemental and isotopic results for batch adsorption-desorption experiments

Batch adsorption-desorption experiments showed that the membrane of the diffusion cell held a certain amount of dissolved Mg during the diffusion experiments (Appendix A4). The amount of Mg adsorbed to the membrane in a 40 ppm Mg solution was 2.0–2.3 μg for MgSO_4_, 3.2–3.8 μg for MgCl_2_, and 3.5–3.8 μg for Mg(NO_3_)_2_, regardless of the MWCO of the membrane. The adsorbed MgSO_4_ and MgCl_2_ are isotopically lighter than the bulk Mg solution by 0.13‰ ± 0.17‰ (2SD, *n* = 2) and 0.11‰ ± 0.06‰ (2SD, *n* = 2), respectively; whereas the adsorbed Mg(NO_3_)_2_ is almost isotopically identical to the bulk Mg solution, with a different of 0.02‰ ± 0.02‰ (2SD, *n* = 2) in δ^26^Mg.

### 4.5 Mg speciation calculation results

**Fig. 4:**
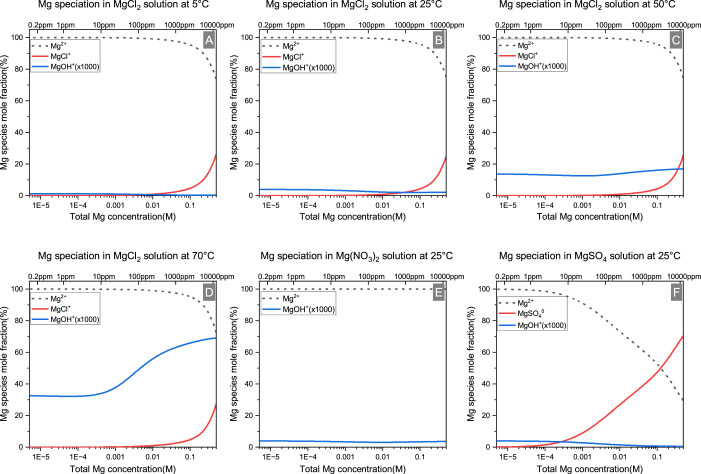
**Speciation of Mg in MgCl**_**2**_**, Mg(NO**_**3**_**)**_**2**_**, and MgSO**_**4**_**solutions under different temperatures and Mg concentrations**.

Speciation of Mg^2+^ ions in the aqueous solutions for the experiments calculated using PHREEQC is summarized in Fig. 4. For MgCl_2_ solutions, > 95% of Mg ions are free Mg^2+^ aquo ion when Mg concentrations are below 0.1 M (2400 ppm), regardless of solution temperature (Fig. 4A–4D). MgCl^+^ is the second most abundant Mg species that complements free Mg^2+^ aquo ion in MgCl_2_ solutions, and its proportion reaches 25% in the initial MgCl_2_ solution that contains 0.5 M (12,000 ppm) Mg. However, it should be noted that the proportion of MgCl^+^ decreases rapidly with decreasing Mg concentration; when Mg concentration is below 0.1 M (2400 ppm), less than 5% of the Mg ions are MgCl^+^. The third notable Mg species in MgCl_2_ solution is MgOH^+^, and its abundance is sensitive to temperature, but even at 70 °C where its abundance is the highest, it only takes up less than 0.7% of dissolved Mg in the MgCl_2_ solutions (Fig. 4D, note the scale is ×1000 for MgOH^+^). For Mg(NO_3_)_2_ solution, PHREEQC calculation results show that almost all (> 99.99%) of Mg^2+^ occurs as free Mg^2+^ aquo ions (Fig. 4E).

PHREEQC calculation results suggest that in a 0.5 M MgSO_4_ solution, the most abundant Mg species is MgSO_4_^0^, which takes up 70.5% of dissolved Mg, whereas free Mg^2+^ aquo ions only comprise 29.5% of the dissolved Mg. The proportion of MgSO_4_^0^ species in the total dissolved Mg decreases with decreasing MgSO_4_ concentration, following a rough linear correlation with log_10_[Mg_total_] to 9% at 0.001 M. Conversely, the proportion of free Mg^2+^ aquo ions increases with log_10_[Mg_total_] to 91% in a 0.001 M (24 ppm) MgSO_4_ solution, and the proportion of free Mg^2+^ aquo ions is even more close to 100% at lower MgSO_4_ concentrations.

## 5 Discussion

### 5.1 Determination of kinetic isotope fractionation factors by diffusion cell method

In the diffusion experiments, the Mg solution in the tubes became progressively enriched in heavy Mg isotopes following a Rayleigh trend (Fig. 3). This reflects that light Mg isotopes preferentially diffused through the membrane to enter the tube over heavy Mg isotopes, as a result, the Mg remaining in the diffusion cell became isotopically heavier with time, and so was the Mg in the subsequent tubes. Therefore, diffusion of Mg ions in aqueous solution is accompanied with negative Mg isotope fractionation, that is, D_26Mg_/D_24Mg_ (α) < 1. It should be noted that the data points in Fig. 3 started to deviate from a linearly increasing trend when the Mg concentration in the tube was below 3–5 ppm. This could be due to the isotopic effect of the interaction between the membrane and aqueous Mg such as adsorption of Mg ions onto the membrane surface, which is discussed in the following subsection.

**Fig. 5:**
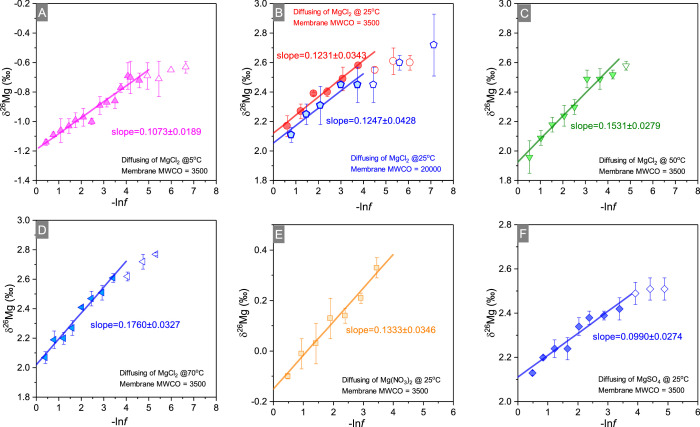
**Plots of δ^26^Mg versus -ln*f* for different experiments**. Vertical error bar represent 2 standard deviation of multiple isotope analysis. Linear regression of the filled data points (error weighted) were performed using an Origin® software, and the uncertainty of the slope represent 1 standard error. The empty data points were excluded for regression, for details see Section 5.1 of the text.

**Fig. 6:**
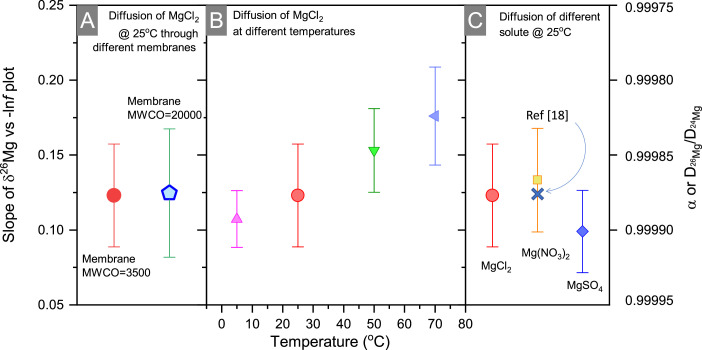
**Summary of the slope in plot of δ^26^Mg versus -ln*f* for different experiments (as shown in**Fig. 5**), note the slope data correspond to α (D_26Mg_/D_24Mg_, left vertical axis)**. The error bars represent the fully propagated uncertainties from the regressions in Fig. 5, incorporating uncertainties in both the Mg isotope ratios and *f*. The α for Mg(NO_3_)_2_ determined by Li et al. [18] is shown as cross in Fig. 6C for comparison.

The δ^26^Mg values of Mg solutions in tubes are plotted against -ln*f* for each experiment in Fig. 5. Similar to Fig. 3, the data points in Fig. 5 also follow a linear trend except for low *f* samples (corresponding to Mg concentration below 3–5 ppm). Importantly, Eq. 5 shows that the slope of the data trend in such plot (*δ_tube_* versus -ln*f*) equals (1-D*/D)*1000, which is a direct link to the kinetic isotope fractionation factor (D*/D, or α) associated with chemical diffusion. Thus, the Mg isotope fractionation factors associated with diffusion under various conditions are determined by linear regression of the data (low *f* samples excluded) in Fig. 5 using an Origin® software, and the results show that α (D_26Mg_/D_24Mg_) varied from 0.99982 ± 0.00003 to 0.99990 ± 0.00003 in the different diffusion experiments (Table 1). In Fig. 6, the slope and corresponding α values for the different experiments are summarized. There is a strong correlation between α (D_26Mg_/D_24Mg_; or slope of plots in Fig. 5) and the experimental temperature, and α for MgSO_4_ is notably more close to 1 than those of MgCl_2_ and Mg(NO_3_)_2_ under the same experimental conditions. The measured α values for MgCl_2_ and Mg(NO_3_)_2_ in this study are indistinguishable within errors, and are also consistent with that reported by Li et al. [18] for Mg(NO_3_)_2_ based on a slightly different “diffusion cell” setup (Table 1; Fig. 6).

**Table 1:** **Summary of the slope data for the δ**^**26**^**Mg vs. -ln*****f*****plots in**Fig. 5**, and the corresponding D**_**26Mg**_**/D**_**24Mg**_**and β factors for Mg in this study, with a comparison of β factors for Mg and other elements in literature**.

Solute /element	r_M-O_^a^(pm)	Temp ( °C)	Membrane MWCO (dalton)	slope	1 σ	D_26Mg_/D_24Mg_ (α)	1 σ	β	1 σ	Source
MgCl_2_	208	5	3500	0.1073	0.0189	0.999893	0.000019	0.00134	0.00024	this study
MgCl_2_	208	25	3500	0.1231	0.0343	0.999877	0.000034	0.00154	0.00043	this study
MgCl_2_	208	25	20,000	0.1247	0.0428	0.999875	0.000043	0.00156	0.00053	this study
MgCl_2_	208	50	3500	0.1531	0.0279	0.999847	0.000028	0.00191	0.00035	this study
MgCl_2_	208	70	3500	0.1760	0.0327	0.999824	0.000033	0.00220	0.00041	this study
Mg(NO_3_)_2_	208	25	3500	0.1333	0.0346	0.999867	0.000035	0.00166	0.00043	this study
MgSO_4_	208	25	3500	0.0990	0.0274	0.999901	0.000027	0.00124	0.00034	this study
MgCl_2_ into HCl	208	25	3500	0.1209	0.0145	0.999879	0.000015	0.00151	0.00018	this study
Ca	240	75						0.0045		(Bourg et al., 2010); ref [2]
Fe	213	20						0.0024		(Rodushkin et al., 2004); ref [20]
Zn	208	20						0.0019		(Rodushkin et al., 2004); ref [20]
Ba	290	10						0.01		(van Zuilen et al., 2016); ref [35]
Mg (Mg(NO_3_)_2_)	208	25	3500	0.1230		0.999877		0.00154		(Li et al., 2024); ref [18]
Ca	240	25	3500					0.00636		(Li et al., 2024); ref [18]
Sr	260	25	3500					0.00953		(Li et al., 2024); ref [18]
Ba	290	25	3500					0.01292		(Li et al., 2024); ref [18]

a Data are from Ohtaki and Radnai (1993).

### 5.2 Potential effects of speciation, adsorption, and cation-anion interaction on mg isotope fraction during the diffusion experiments

Experimental results show that significant Mg isotope fractionation occurred during the diffusion experiments (Figs. 3, 5), and the diffusional Mg isotope fractionation factors show systematic correlations with temperature and type of anions (Fig. 6). However, before further discussions on such correlations, the potential isotopic effects of several processes associated with the diffusion experiments must be carefully evaluated. These processes include Mg speciation in the fluid, Mg adsorption on the membrane, and cation-anion interaction across the diffusional boundary, which are discussed separately as below.

#### 5.2.1 Speciation of mg in aqueous solutions and the isotopic effects

Results of PHREEQC calculation show that for MgCl_2_ and Mg(NO_3_)_2_ solutions, free Mg^2+^ aquo ion is the dominant Mg species under the experimental conditions (Fig. 4). Mg(NO_3_)_2_ solutions are essentially unaffected by effects of aqueous speciation for Mg (Fig. 4e), and the difference in Mg speciation across the membrane is also insignificant for MgCl_2_ solutions with Mg concentration below 0.1 M (Fig. 4a–d). Furthermore, according to the calculation of Schott et al. [28], the reduced partition function ratios (RPFR, or β factor) of free Mg^2+^ aquo ion and MgCl^+^ aquo ion are very close, with a difference less than 0.07 ‰ for ^26^Mg/^24^Mg at 25 °C. Accordingly, even for a 0.5 M MgCl_2_ solution, the inter-species Mg isotope fractionation will only cause an offset of 0.02 ‰ to the δ^26^Mg of free Mg^2+^ aquo ions relative to a dilute MgCl_2_ solution based on isotope mass balance. This offset is well within the analytical uncertainty and is thus negligible.

By contrast, speciation of Mg^2+^ ions in MgSO_4_ solutions is variable and highly dependent on MgSO_4_ concentration (Fig. 5F). Theoretical calculations suggest significant Mg isotope fractionation between MgSO_4_^0^ and Mg^2+^ aquo ions, with Δ^26^Mg_MgSO4 - Mg2+_ (i.e., β_MgSO4_ - β_Mg2+_) varying from −0.13 ‰ for outer-sphere complex of MgSO_4_^0^ [29] and 2.62‰ for inner-sphere complex of MgSO_4_^0^ [28]. It has been suggested that 7%−8% of dissolved Mg occurs as inner-sphere complex of MgSO_4_^0^ in > 0.1 M MgSO_4_ solutions [30]. Therefore, inter-species Mg isotope fractionation in MgSO_4_ solutions could be significant to superimpose on the kinetic isotope effect during diffusion driven by Mg concentration gradient.

#### 5.2.2 Adsorption of mg by membrane and the isotopic effect

Batch adsorption-desorption experiments of the cell membranes in solutions that contained 40 ppm Mg show that the mass of adsorbed Mg was on the order of 2–4 μg, with Mg isotope fractionation factors of 0–0.1‰ (Appendix A4). Therefore, the effect of Mg adsorption to membrane should be negligible at the early stage of diffusion experiment because the flux of Mg driven by chemical diffusion overwhelmed the Mg that was adsorbed to the membrane. However, during the late-stage of the diffusion experiments, when Mg contents in the cell and the tube were sufficiently low, the adsorbed Mg on the membrane could be significant compared with the flux of aqueous Mg^2+^ that diffused through the membrane pores, and the adsorption-related Mg isotope fractionation could interfere the diffusion-driven Mg isotope fractionation signatures. In this case, the δ^26^Mg of Mg in the diffusion tube would no longer follow a simple Rayleigh distillation trend, and the data points in a plot of δ^26^Mg versus -ln*f* would start to deviate from a linear trend. Indeed, this is what has been observed in most diffusion cell experiments, where the δ^26^Mg of solutions leveled off at -ln*f* of around 4 (i.e., Mg concentration in diffusion tube was below 3–5 ppm). Thus, the data points of low Mg content solutions deviating from the linear trend of the diffusion experiments was not considered for calculation of diffusion related Mg isotope fractionation factors, as shown in Fig. 5.

#### 5.2.3 Effect of cation-anion interaction during diffusion

**Fig. 7:**
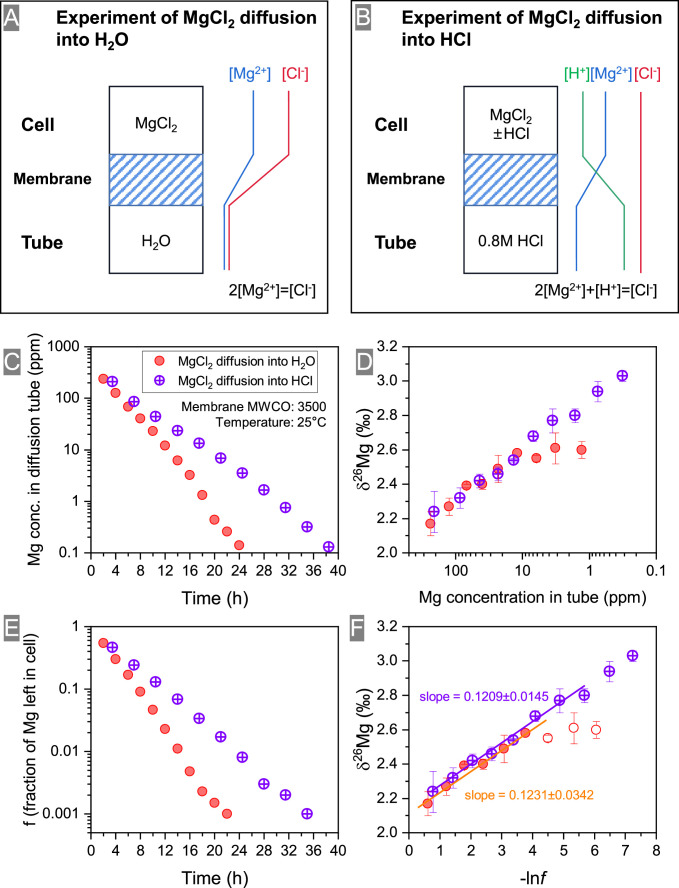
**Comparison of MgCl_2_ diffusion experiments under the same temperature (25 °C) and through the same membrane (MWCO=3500), but with different ion balance conditions**. In a set of experiment (Fig. 7A), the MgCl_2_ solution was diffused into H_2_O, where concentration gradient existed for both Mg^2+^ and Cl^−^. In another set of experiment (Fig. 7B), 0.4 M MgCl_2_ solution was diffused into 0.8 M HCl, where there was no concentration gradient for Cl^−^, but the concentration gradient of Mg^2+^ was balanced by the diffusion of *H*^+^ at an opposite direction for charge balance. The elemental and isotopic results of the two MgCl_2_ diffusion experiments are compared in plots C to F. (For interpretation of the references to colour in this figure legend, the reader is referred to the web version of this article.)

In most of the experiments in this study, the Mg solutions were diffused into pure water, where the diffusion of Mg was dictated by not only the concentration gradient across the membrane but also the charge balance within the aqueous solution. Take diffusion of MgCl_2_ into H_2_O for example, the Mg^2+^ and Cl^−^ need to diffuse proportionally (i.e., 2×flux_[Mg2+]_ = flux[_Cl-_]) to maintain the charge balance of the solute (Fig. 7A). Note that Mg^2+^ and Cl^−^ have different self-diffusion coefficients, which are 0.706 × 10^−9^ m^2^/s and 2.032 × 10^−9^ m^2^/s at 25 °C, respectively [31], meaning that Cl^−^ would diffuse faster than Mg^2+^ in water in an idealized unconstrained condition. Due to the charge balance constraint, the actual diffusion coefficient of dissolved MgCl_2_ in water is higher than the self-diffusion coefficients of Mg^2+^, but lower than the self-diffusion coefficients of Cl^−^ [32]. This implies that diffusion of Mg^2+^ in the experimental setting of Fig. 7A was accelerated by the “pulling” of Cl^−^ by microscopic cation-anion interaction.

In order to evaluate the potential isotopic effect of cation-anion interaction in solutions during diffusion, we performed a diffusion experiment of MgCl_2_ in a constant [Cl^−^] environment across the membrane (Fig. 7B). In this experiment, the diffusion cell contained a 0.4 M MgCl_2_ solution, and the diffusion tubes contained 0.8 M HCl; therefore, there was no concentration gradient in [Cl^−^], and the charge balance of Mg^2+^ diffusion was maintained by compensation of counter-diffusion of *H*^+^ from the tube into the cell (Fig. 7B). In this case, Mg^2+^ diffusion should not be affected by cation-anion interaction as in the case of Fig. 7A, and due to the lack of “pulling” by Cl^−^, the diffusion of Mg^2+^ would be slower than the case of MgCl_2_ diffusion into water. Indeed, this is reflected in the elemental results that show slower decreases in Mg concentration in the diffusion tube (Fig. 7C) as well as *f* (Fig. 7E) in the experiment of MgCl_2_ diffusion into HCl compared to those of MgCl_2_ diffusion into water. However, it is remarkable that Mg isotope fractionation behaviors of the two experiments are very similar, particularly at the initial stage (Fig. 7D), yielding identical slopes in a δ^26^Mg versus -ln*f* plot (Fig. 7F). This suggests that cation-anion interaction affects the elemental behavior during the experiments of Mg salts diffusing into water, but it may not affect the Mg isotope fractionation factors compared to scenarios without counter ion gradient.

Based on the above discussion, we conclude that adsorption on the membrane and cation-anion interaction do not affect the diffusion-driven Mg isotope fractionation in the experiments, but the effect of Mg^2+^ speciation with SO_4_^2−^ cannot be ignored. Thus the observed differences in Mg isotope fractionation factors in the different diffusion experiments could be used to reveal the intrinsic physical-chemical controls of kinetic Mg isotope fractionation in aqueous solutions.

### 5.3 The mass dependence Mg isotope fractionation during diffusion in aqueous solutions and its controlling factors

Contrary to the previous study that suggested no discernable Mg isotope fractionation [3], our experiments proved the existence of Mg isotope fractionation during chemical diffusion of Mg ions in aqueous solutions. That is, diffusion of Mg ions in aqueous solutions is not completely mass independent, but follows an equation of

(6) $$\frac{D_{H}}{D_{L}}={\left(\frac{M_{L}}{M_{H}}\right)}^{\beta }$$

where M_L_ and M_H_ are the masses of the light and heavy isotopes of interest, respectively, and β is an empirical constant that can be used to quantify the mass dependence of diffusion coefficients for the system [3]. Under one end-member scenario of diffusion of ideal gas, the diffusion coefficient D is proportional to the velocity of the particle, which is inversely proportional to the square root of the mass of the particle following a kinetic theory, thus β = 0.5. By contrast, under the other end-member scenario, the diffusion coefficient D is completely unrelated to the mass of the diffusion particle (β equals to 0), but is only dependent on the bulk fluid physical property following Fick’s law of diffusion, which is termed as hydrodynamic [33]. During diffusion in aqueous solutions, the β values of different species vary between 0 and 0.5, which is indicative of the intermediate behaviors of the diffusing species between the two extreme scenarios [2,3,33]. Thus β value allows a comparison of the mass dependence of chemical diffusion between different elements, which provides insights into the physical behavior of the diffusing species in aqueous solutions at the molecular level.

**Fig. 8:**
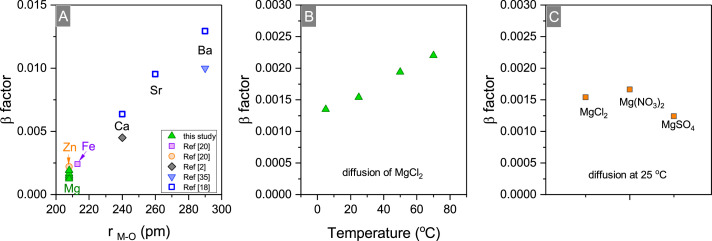
A. Summary β factors of aqueous Mg measured in this study and β factors of other divalent elements. B. β factors of aqueous Mg as a function of different temperature. C. Comparison of β factors of aqueous Mg in different salt solutions. (For interpretation of the references to colour in this figure legend, the reader is referred to the web version of this article.)

The β values for various divalent elements during diffusion in aqueous solution are compiled in Table 1 and plotted in Fig. 8. As shown in Fig. 8A, the β values of the divalent elements are below 0.01, and there appears to be a general trend of increasing β with increasing r_M-O_, where r_M-O_ is the average ion-water distance for the first hydration shell of ions [20,34,35]. This implies that the diffusion behavior of the ions is more mass dependent with longer ion-water distance. Given the same charge of the ion, shorter r_M-O_ would result in higher surface charge density for the aquo ion, which would induce greater Coulombic force between the aquo ion and the surrounding free water molecules, thus there would be stronger hydrodynamic friction for the ions with shorter r_M-O_ and the diffusion coefficient would be less mass dependent. Another reason for the correlation between β and r_M-O_ within the same element group is the rigidity of the hydration shell for the aquo ion. The bond strength between an ion and the water molecule in the first hydration shell is weaker for longer r_M-O_, and the residence time of water molecule in the first hydration shell would be shorter. A strong correlation between β and the inverse of the residence time of water molecule in the first hydration shell of elements has been reported by [2].

Experimental results in this study (Fig. 8B) show that β for MgCl_2_ increases with increasing temperature (i.e., from 0.00135 at 5 °C to 0.00220 at 70 °C), pointing to a minor, but significant control of temperature on β for diffusing ions in aqueous solution. Similar positive correlation of β and temperature has also been observed in diffusion experiments for Cl isotopes [19]. The temperature dependence of β could also be explained by ion-water interaction. First, the viscosity of water decreases with increasing temperature [36], meaning that the molecular-level friction caused by hydrogen bond between water molecules decreases with increasing temperature. Consequently, the movement of aquo ions in aqueous solution is less hindered by molecular frictions at higher temperatures, and the diffusional behavior of the element becomes less hydrodynamic but more kinetic [33], thus β increases with increasing temperature. Second, water exchange rate in the first hydration shell of Mg^2+^ decreases with increasing temperature [37], because β shows strong correlation with the inverse of the residence time of water molecules in the first hydration shell in aqueous solutions [2], increasing temperature will lead to the increase of β for ions.

Our experiments also show that mass dependence of isotope fractionation during ion diffusion could be influenced by the counter-ion in the aqueous solution, as β factor for MgSO_4_ is lower than those of MgCl_2_ and Mg(NO_3_)_2_ under the same experimental condition (Fig. 8C). It should be noted that the rate of diffusional loss of MgSO_4_ through the membrane is also remarkably lower than those of MgCl_2_ and Mg(NO_3_)_2_ (Fig. 2C). This implies that at atomic scale, Mg^2+^ ions in a MgSO_4_ aqueous solution receives more molecular friction, which leads to more hydrodynamic nature of the diffusion process. Indeed, Mg^2+^ and SO_4_^2−^ tend to form ion-pairs (MgSO_4_^0^, Fig. 4), and both Mg^2+^ and SO_4_^2−^ are classified as strong “structure makers” for aqueous solution as their existence can increase the viscosity of solution by enhancing the hydrogen bond network surrounding the ions in the aqueous solution [38]. Therefore, the influence of anions on β factor of Mg^2+^ in aqueous solutions also reflects the strength of ion-water interaction at molecular scale.

### 5.4 Implications

The experimental results of this study show that diffusion of aqueous Mg driven by concentration gradient is ubiquitously associated with a measurable kinetic Mg isotope fractionation, confirming the report of Li et al. [18]. It is necessary to re-evaluate the effect of once omitted kinetic Mg isotope fractionation in aqueous solutions on interpretations of Mg isotope data observed from natural samples. One prominent case is the interpretation of Mg isotope data from biogenic magnesian calcite. It has long been recognized that calcite skeletons of some marine organisms (such as planktonic foraminifera) have very low δ^26^Mg values, which can be as low as −5.5‰, corresponding to an isotopic offset of −4.7‰ relative to seawater [39,40]. Such Mg isotopic offset is much lower than the experimentally determined Mg isotope fractionation between calcite and aqueous solutions [i.e., −2.5‰ to −3.5‰ at 25 °C]; [[25], [41], [42], [43]], a problem remaining unsolved till the present time. Hippler et al. [39] raised the possibility of diffusion-induced Mg isotope fractionation in interpreting the extremely low δ^26^Mg signatures of biogenic calcite. Indeed, foraminifera actively pump Mg^2+^ out of their cells [44], creating a chemical gradient of Mg across their membranes, which could cause diffusion driven Mg isotope fractionation. Our study indicates that diffusion can only induce a 0.1‰−0.2‰ decrease in δ^26^Mg of the in-cell fluid relative to seawater outside the cell water, due to the limited diffusion-related Mg isotope fractionation factor (D_26Mg_/D_24Mg_ = 0.99982 to 0.99990). Therefore, although diffusion of Mg ions through cell walls of organisms could be a contributing factor to the low δ^26^Mg signatures of biogenic calcite, the low δ^26^Mg signatures of biogenic calcite in certain marine calcifiers cannot be fully explained by diffusion through cell walls of the organisms, and other mechanisms such as vital effects might play the major role in enhancing preferential incorporation of light Mg isotopes into these biogenic calcites.

Elemental concentration gradient of Mg is commonly developed in pore water of soft sediments in seafloor, where aqueous Mg is consumed by clay formation and dolomitization during early diagenesis. Remarkable Mg isotope variability exists in pore water of sediment cores of Ocean Drilling Program, and the pattern of Mg isotope variation along the drill core differs greatly between different drilling sites; in some drill cores, δ^26^Mg of pore water increases downward with decreasing Mg concentration, whereas in other drill cores, δ^26^Mg of pore water decreases downward with decreasing Mg concentration [45]. It has been suggested that clay formation would result in downward-decreasing trend of δ^26^Mg in pore water for three drill cores, and based on fitting of data by numeric models, they derived the Mg isotope fractionation factors (Δ^26^Mg_clay-sw_) for clay formation in these drill cores, which ranges from +0.1‰ to +1.2‰ [45]. The diffusion-driven Mg isotope fractionation factor for aqueous Mg (α = 0.99982 to 0.99990) is on the same order of magnitude with Δ^26^Mg_clay-sw_, which is non-negligible. It is important to note that, in almost all published studies that utilized reaction-transport models to understand the behaviors of Mg isotopes in carbonates during diagenesis [[45], [46], [47], [48]], the diffusion-related Mg isotope effect was not taken into consideration. We therefore urge future studies of this type to incorporate the diffusion-related Mg isotope fractionation factor in numeric models for more accurate modeling outcomes.

The experimental results in this study also provide new insights for understanding and prediction of kinetic isotope fractionation in fluids over a broader physico-chemical conditions. Our study shows that, kinetic isotope fractionation of elements driven by chemical diffusion in aqueous solution would be more prominent at higher temperatures, which is related to the lower viscosity of the fluid and the underlying mechanism of weaker hydration “cage” and hydrogen bond network surrounding the ion. This is in stark contrast to equilibrium isotope partitioning between different phases, which decreases in magnitude with increasing temperature [49,50]. Based on the relationship between β factors and the ion’s valence, r_M-O_, temperature, and property of counter ions as discussed in the previous section, we speculate that chemical diffusion in supercritical fluids would be accompanied with significant kinetic isotope fractionation, because supercritical fluids have low viscosity, weak hydrogen bonds, and high chance of neutral ion-pair formation [51], in which the movement of ions would show more kinetic rather than hydrodynamic behavior. Dehydration during subduction of volatile-rich materials, and late-magmatic to hydrothermal stages of plutonic magmatism are the potential processes that could be associated with supercritical fluid activity, and kinetic isotope fractionation could have contributed to the abnormal metal stable isotope compositions reported in recent studies of eclogites [52], pegmatites [53], and porphyry deposits [54].

## 6 Conclusion

In this contribution, we modified the recently developed “diffusion cell” method to investigate the behaviors of Mg isotopes during diffusion in aqueous solution under various conditions. In all experiments, the Mg remaining in the diffusion cell became progressively isotopically heavier, suggesting that light Mg isotopes preferentially diffused out of the cell through the membrane relative to heavy Mg isotopes. This attests to the existence of kinetic Mg isotope fractionation during chemical diffusion in aqueous solution. The diffusional kinetic Mg isotope fractionation factors (α, or D_26Mg_/D_24Mg_) are not controlled by the pore size of the membrane of the diffusion cell, but are correlated with temperature, ranging from 0.99989 at 5 °C to 0.99982 at 70 °C, showing that the magnitude of kinetic isotope fractionation associated with chemical diffusion in aqueous solution increases with temperature. The α factor for Mg is not affected by the cation-anion interaction between Mg^2+^ and Cl^−^ or Mg^2+^ and NO_3_^−^, but diffusion of MgSO_4_ shows notably less significant isotope fractionation relative to those of MgCl_2_ and Mg(NO_3_)_2_, (i.e., 0.99992 for MgSO_4_ vs 0.99986 for Mg(NO_3_)_2_ and 0.99989 for MgCl_2_ at 25 °C).

The mass dependence of kinetic isotope fractionation for aqueous Mg is compared with other elements through a parameter β. A comparison of all available β factors for different divalent elements shows that the mass dependence of kinetic isotope fractionation is primarily controlled by atomic distance between the ion and the neighboring water molecules, but also affected by temperature and nature of counter ions. In general, elements with longer ion-water distance for the aqueous species show more significant mass-dependent kinetic isotope fractionation during diffusion, and diffusion-driven isotope fractionation in aqueous solutions is more significant under higher temperatures, and without the presence of SO_4_^2−^. We therefore reason that mass dependence of kinetic isotope fractionation of elements during diffusion in aqueous solution is fundamentally dependent on the ion-water interaction intensity; a stronger ion-water interaction would exert stronger molecular friction on the aquo ion and result in more significant hydrodynamic nature of the movement of the ion, which leads to smaller mass dependence of kinetic isotope fractionation, and vice versa.

## Declaration of competing interests

The authors declare that they have no conflicts of interest in this work.

## Appendix Supplementary materials

Image, application 1

Image, application 2

Image, application 3

Video showing how the custom-made robotic arm was working for the diffusion cell experiment, note the time for tube switching is dramatically shortened for demonstration purpose. Image, video 4 dummy alt text
