# Supplementary material for: Kinetic fractionation of Mg isotopes during chemical diffusion in aqueous solutions: A reappraisal
Source: Fundam Res. 2024 Dec 25;6(4):2387–97. doi: 10.1016/j.fmre.2024.11.010 (PMC13424388; doi:10.1016/j.fmre.2024.11.010)
Supplement: Supplementary file 1 [file mmc1.docx]

**Appendix A1:** Detailed derivation of Equation 5 in the main text from Equations 1-4.

Part 1: A brief summary of Equations 1-4 in the main text

According to Fick’s first law, the diffusional flux (*J*) across the membrane follows:

*J=-DK(C_cell_-C_tube_)*  (Equation 1)

A time integration leads to

$\text{C}_{cell}^{i}/\text{C}_{cell}^{0}=e^{-DKt}=f$ (Equation 2)

And *f* can be calculated by

$f=\frac{\sum_{\text{i}+1}^{n} \text{(C}_{\text{tube}}^{\text{i}+1}\times\text{V}_{\text{tube}})+\text{ C}_{\text{cell}}^{\text{n}}\times\text{V}_{\text{cell}}}{\text{C}_{\text{cell}}^{0}\times\text{V}_{\text{cell}}}=\frac{\sum_{\text{i}+1}^{n} \text{(C}_{\text{tube}}^{\text{i}+1}\times\text{V}_{\text{tube}})+\text{ C}_{\text{cell}}^{\text{n}}\times\text{V}_{\text{cell}}}{\sum_{1}^{n} \text{(C}_{\text{tube}}^{\text{i}}\times\text{V}_{\text{tube}})+\text{ C}_{\text{cell}}^{\text{n}}\times\text{V}_{\text{cell}}}$ (Equation 3)

where V is the volume of aqueous solution in the tube or cell.

If we consider diffusion of two isotopes (*l* and *h*) in the system, which have diffusion coefficient of *D* and *D**, respectively. The evolution of isotopic composition (R) within the cell can be expressed as

$\frac{\text{R}_{cell}^{\text{i}}}{\text{R}_{cell}^{0}}=\frac{C_{cell}^{h,i}/C_{cell}^{l,i}}{C_{cell}^{h,0}/C_{cell}^{l,0}}=\left(\frac{C_{cell}^{h,i}}{C_{cell}^{h,0}}\right)/\left(\frac{C_{cell}^{l,i}}{C_{cell}^{l,0}}\right)$ (Equation 4)

In Equation 5, the isotope composition of solute in the diffusion tube (δ_tube_) is linearly correlated with ln*f*, that

$\delta_{tube}^{i}=\ln f\cdot(D^{*}/D-1)\cdot1000+(\delta_{cell}^{0}+k)$ (Equation 5)

Part 2: Detailed derivation of Equation 5 from Equations 1-4, following the techniques in Richter (2006).

Plug Equation 2 into Equation4, we have

$\frac{\text{R}_{cell}^{\text{i}}}{\text{R}_{cell}^{0}}=e^{-(D^{*}-D)Kt}=e^{-DKt(D^{*}/D-1)}=\frac{C_{cell}^{i}}{C_{cell}^{0}}e^{(D^{*}/D-1)}$ (Equation 6)

Take the natural log for both sides, we have

$\ln(R_{cell}^{i}/R_{cell}^{0})=\ln\left(C_{cell}^{i}/C_{cell}^{0}\right)\cdot(D^{*}/D-1)$ (Equation 7)

The left side of Equation 7 can be rearranged to

$\ln(R_{cell}^{i}/R_{cell}^{0})=\ln(R_{cell}^{i}/R_{std})-\ln(R_{cell}^{0}/R_{std})$ (Equation 8)

where *R_std_* is the isotope ratio of the standard, based on the definition of delta notation for stable isotopes (δ=(R/R_std_-1)*1000) and that ln(R/R^*^) ≈ R/R^*^-1 (when R/R* is very close to 1, as the case of isotope partitioning), Equation 7 can be rewritten as

$\delta_{cell}^{i}-\delta_{cell}^{0}\approx\ln f\cdot(D^{*}/D-1)\cdot1000$ (Equation 9)

Equation 9 shows that, in a plot of *δ* versus ln*f*, the data points for solutes in the diffusion cell plot along a linear trend, which has a slope that corresponds to the kinetic isotope fractionation factor associated with diffusion (*D^*^/D*).

Now we consider the isotopic composition of solute within the outer centrifuge tube (δ_tube_), which accumulated the Mg diffused out of the membrane over a uniform period of time (Δt) in the diffusion experiments. Assuming that the diffusion cell had been put into *i* tubes, the isotopic compositions of solute in the cell before and after diffusion experiment in the *(i+1)^th^* tube are $\delta_{cell}^{i}$ and $\delta_{cell}^{i+1}$ respectively. Based on elemental and isotopic mass balance,

$V_{cell}\cdot C_{cell}^{i}=V_{cell}\cdot C_{cell}^{i+1}+V_{tube}\cdot C_{tube}^{i+1}$ (Equation 10)

$\delta_{cell}^{i}\cdot V_{cell}\cdot C_{cell}^{i}=\delta_{cell}^{i+1}\cdot V_{cell}\cdot C_{cell}^{i+1}+\delta_{tube}^{i+1}\cdot V_{tube}\cdot C_{tube}^{i+1}$ (Equation 11)

Equation 2 indicates that $C_{cell}^{i+1}/C_{cell}^{i}$(defined here as *f’*) is a constant if the diffusion cell stayed equal amount of time in each diffusion tube (which is the case in our experiment). Plug Equations 2, 9, and 10 into Equation 11 and rearrange, we have

$\delta_{tube}^{i+1}=\delta_{cell}^{i+1}-\ln f'/(1-f')\cdot(D^{*}/D-1)\cdot1000=\delta_{cell}^{i+1}+k$ (Equation 12)

Equation 12 shows that there is a constant isotopic offset ($k=-1000\cdot\left(D^{*}/D-1\right)\cdot\ln f'/(1-f')$) between the solutions in the cell and tube after each stage of diffusion in the tube. Therefore, by combining Equation 9 and Equation 12, we have

$\delta_{tube}^{i}=\ln f\cdot(D^{*}/D-1)\cdot1000+(\delta_{cell}^{0}+k)$ (Equation 13)

Equation 13 (the Equation 5 in the main text) shows that, in a plot of *δ* versus ln*f*, δ_tube_ also plots along a linear trend that is parallel with the data trend for δ_cell_. Therefore, the kinetic isotope fractionation factor associated with diffusion (*D^*^/D*) can also be derived by systematic analysis of isotopic composition of the solutions in the tubes in a diffusion experiment.
