# Supplementary material for: Kinetic fractionation of Mg isotopes during chemical diffusion in aqueous solutions: A reappraisal
Source: Fundam Res. 2024 Dec 25;6(4):2387–97. doi: 10.1016/j.fmre.2024.11.010 (PMC13424388; doi:10.1016/j.fmre.2024.11.010)
Supplement: Supplementary file 2 [file mmc2.docx]

**Appendix A3.** Elemental and isotopic data for the different diffusion experiments

Table A3-1: Diffusion experiment for MgCl_2_ at 5°C, through membrane of MWCO=3500 towards deionized water.

| **Sample**  **No.** | **Time**  **(hours)** | **Mg conc.**  **(ppm)** | **Mg mass**  **(ug)** | **f** | **-lnf** | **lnf-err** | **d26Mg** | **2SD** | **d25Mg** | **2SD** | **n** |
| --- | --- | --- | --- | --- | --- | --- | --- | --- | --- | --- | --- |
|  |  |  |  |  |  |  |  |  |  |  |  |
| Tube-1 | 2 | 218.0 | 10899 | 0.648 | 0.434 | 0.075 | -1.14 | 0.02 | -0.54 | 0.04 | 3 |
| Tube-2 | 4 | 106.1 | 5307 | 0.471 | 0.753 | 0.074 | -1.09 | 0.02 | -0.52 | 0.07 | 3 |
| Tube-3 | 6 | 80.3 | 4013 | 0.338 | 1.086 | 0.074 | -1.06 | 0.08 | -0.52 | 0.06 | 3 |
| Tube-4 | 8 | 62.0 | 3098 | 0.235 | 1.450 | 0.076 | -1.03 | 0.05 | -0.49 | 0.05 | 3 |
| Tube-5 | 10 | 35.5 | 1773 | 0.176 | 1.740 | 0.073 | -0.99 | 0.05 | -0.46 | 0.04 | 3 |
| Tube-6 | 12 | 30.5 | 1523 | 0.125 | 2.080 | 0.073 | -0.97 | 0.06 | -0.48 | 0.08 | 3 |
| Tube-7 | 14 | 23.4 | 1168 | 0.086 | 2.453 | 0.074 | -1.00 | 0.02 | -0.47 | 0.02 | 3 |
| Tube-8 | 16 | 16.1 | 807 | 0.059 | 2.827 | 0.076 | -0.89 | 0.06 | -0.43 | 0.03 | 3 |
| Tube-9 | 18 | 9.4 | 472 | 0.044 | 3.135 | 0.076 | -0.87 | 0.03 | -0.42 | 0.02 | 3 |
| Tube-10 | 20 | 7.9 | 396 | 0.030 | 3.497 | 0.080 | -0.86 | 0.05 | -0.41 | 0.04 | 3 |
| Tube-11 | 22 | 4.3 | 213 | 0.023 | 3.759 | 0.079 | -0.77 | 0.03 | -0.39 | 0.03 | 3 |
| Tube-12 | 24 | 3.7 | 185 | 0.017 | 4.069 | 0.081 | -0.69 | 0.09 | -0.33 | 0.07 | 3 |
| Tube-13 | 26 | 1.0 | 51 | 0.015 | 4.173 | 0.069 | -0.70 | 0.11 | -0.32 | 0.07 | 3 |
| Tube-14 | 28 | 3.1 | 153 | 0.010 | 4.576 | 0.068 | -0.72 | 0.05 | -0.35 | 0.07 | 3 |
| Tube-15 | 30 | 1.8 | 92 | 0.007 | 4.930 | 0.063 | -0.69 | 0.09 | -0.33 | 0.09 | 3 |
| Tube-16 | 32 | 1.7 | 87 | 0.004 | 5.440 | 0.061 | -0.71 | 0.12 | -0.34 | 0.07 | 3 |
| Tube-17 | 34 | 1.1 | 56 | 0.002 | 5.999 | 0.058 | -0.65 | 0.00 | -0.32 | 0.04 | 3 |
| Tube-18 | 36 | 0.7 | 36 | 0.001 | 6.663 | 0.051 | -0.63 | 0.04 | -0.29 | 0.06 | 3 |
| Tube-19 | 38 | 0.8 | 38 | 0.000 |  |  | -0.57 | 0.03 | -0.26 | 0.03 | 3 |
| Cell-final |  | NA |  |  |  |  |  |  |  |  |  |

Table A3-2: Diffusion experiment for MgCl_2_ at 25°C, through membrane of MWCO=3500 towards deionized water.

| **Sample**  **No.** | **Time**  **(hours)** | **Mg conc.**  **(ppm)** | **Mg mass**  **(ug)** | **f** | **-lnf** | **lnf-err** | **d26Mg** | **2SD** | **d25Mg** | **2SD** | **n** |
| --- | --- | --- | --- | --- | --- | --- | --- | --- | --- | --- | --- |
|  | 0 |  |  |  |  |  | 2.23 | 0.05 | 1.16 | 0.06 | 4 |
| Tube-1 | 2 | 237.7 | 11886 | 0.541 | 0.613 | 0.062 | 2.17 | 0.07 | 1.10 | 0.02 | 3 |
| Tube-2 | 4 | 125.4 | 6272 | 0.300 | 1.205 | 0.062 | 2.27 | 0.05 | 1.16 | 0.02 | 3 |
| Tube-3 | 6 | 68.2 | 3412 | 0.168 | 1.784 | 0.061 | 2.39 | 0.01 | 1.24 | 0.01 | 3 |
| Tube-4 | 8 | 40.0 | 2001 | 0.091 | 2.399 | 0.060 | 2.40 | 0.03 | 1.24 | 0.02 | 3 |
| Tube-5 | 10 | 23.1 | 1155 | 0.046 | 3.074 | 0.059 | 2.49 | 0.08 | 1.27 | 0.09 | 3 |
| Tube-6 | 12 | 12.1 | 603 | 0.023 | 3.773 | 0.059 | 2.58 | 0.00 | 1.32 | 0.03 | 3 |
| Tube-7 | 14 | 6.1 | 307 | 0.011 | 4.496 | 0.058 | 2.55 | 0.02 | 1.29 | 0.01 | 3 |
| Tube-8 | 16 | 3.2 | 162 | 0.005 | 5.320 | 0.061 | 2.61 | 0.09 | 1.34 | 0.02 | 3 |
| Tube-9 | 18 | 1.3 | 65 | 0.002 | 6.044 | 0.067 | 2.60 | 0.05 | 1.34 | 0.03 | 3 |
| Tube-10 | 20 | 0.4 | 22 | 0.001 |  |  |  |  |  |  |  |
| Tube-11 | 22 | 0.3 | 13 | 0.001 |  |  |  |  |  |  |  |
| Tube-12 | 24 | 0.1 | 7 | 0.001 |  |  |  |  |  |  |  |
| Cell-final |  | 9.2 | 18 |  |  |  |  |  |  |  |  |
| Total Mg  Mass (∑) |  |  | 24156 |  |  |  |  |  |  |  |  |

* original Mg mass in diffusion cell was 25500 ug (12750ppm/ml, 2ml), the relative difference between the original Mg mass in diffusion cell and the total Mg mass in diffusion tubes after the diffusion experiment was 5.3%.

Table A3-3: Diffusion experiment for MgCl_2_ at 25°C, through membrane of MWCO=20000 towards deionized water.

| **Sample**  **No.** | **Time**  **(hours)** | **Mg conc.**  **(ppm)** | **Mg mass**  **(ug)** | **f** | **-lnf** | **lnf-err** | **d26Mg** | **2SD** | **d25Mg** | **2SD** | **n** |
| --- | --- | --- | --- | --- | --- | --- | --- | --- | --- | --- | --- |
|  | 0 |  |  |  |  |  | 2.23 | 0.05 | 1.16 | 0.06 | 3 |
| Tube-1 | 2 | 362.04 | 18102 | 0.4539 | 0.790 | 0.060 | 2.11 | 0.05 | 1.09 | 0.07 | 3 |
| Tube-2 | 4 | 147.31 | 7366 | 0.2317 | 1.462 | 0.060 | 2.25 | 0.07 | 1.15 | 0.03 | 3 |
| Tube-3 | 6 | 71.49 | 3574 | 0.1239 | 2.089 | 0.057 | 2.31 | 0.13 | 1.17 | 0.01 | 3 |
| Tube-4 | 8 | 48.98 | 2449 | 0.0500 | 2.996 | 0.058 | 2.45 | 0.02 | 1.24 | 0.01 | 3 |
| Tube-5 | 10 | 17.37 | 868 | 0.0238 | 3.739 | 0.058 | 2.45 | 0.12 | 1.24 | 0.05 | 3 |
| Tube-6 | 12 | 7.92 | 396 | 0.0118 | 4.438 | 0.054 | 2.45 | 0.12 | 1.24 | 0.05 | 3 |
| Tube-7 | 14 | 5.34 | 267 | 0.0038 | 5.580 | 0.053 | 2.60 | 0.05 | 1.32 | 0.03 | 3 |
| Tube-8 | 16 | 1.96 | 98 | 0.0008 | 7.111 | 0.078 | 2.72 | 0.21 | 1.38 | 0.10 | 3 |
| Tube-9 | 18 | 0.50 | 25 | 0.0001 |  |  |  |  |  |  |  |
| Cell-final |  | NA | 0 | 0.0001 |  |  |  |  |  |  |  |
|  |  |  |  |  |  |  |  |  |  |  |  |

Table A3-4: Diffusion experiment for MgCl_2_ at 50°C, through membrane of MWCO=3500 towards deionized water.

| **Sample**  **No.** | **Time**  **(hours)** | **Mg conc.**  **(ppm)** | **Mg mass**  **(ug)** | **f** | **-lnf** | **lnf-err** | **d26Mg** | **2SD** | **d25Mg** | **2SD** | **n** |
| --- | --- | --- | --- | --- | --- | --- | --- | --- | --- | --- | --- |
|  | 0 |  |  |  |  |  | 2.23 | 0.05 | 1.16 | 0.06 | 3 |
| Tube-1 | 1 | 218.0 | 7793 | 0.606 | 0.501 | 0.064 | 1.96 | 0.11 | 1.04 | 0.04 | 3 |
| Tube-2 | 2 | 106.1 | 4772 | 0.364 | 1.011 | 0.064 | 2.09 | 0.05 | 1.07 | 0.07 | 3 |
| Tube-3 | 3 | 80.3 | 2904 | 0.216 | 1.531 | 0.065 | 2.18 | 0.05 | 1.13 | 0.02 | 3 |
| Tube-4 | 4 | 62.0 | 1718 | 0.129 | 2.046 | 0.065 | 2.24 | 0.07 | 1.13 | 0.01 | 3 |
| Tube-5 | 5 | 35.5 | 943 | 0.081 | 2.509 | 0.063 | 2.30 | 0.05 | 1.18 | 0.07 | 3 |
| Tube-6 | 6 | 30.5 | 690 | 0.046 | 3.072 | 0.063 | 2.49 | 0.06 | 1.24 | 0.02 | 3 |
| Tube-7 | 7 | 23.4 | 398 | 0.026 | 3.644 | 0.063 | 2.49 | 0.07 | 1.26 | 0.02 | 3 |
| Tube-8 | 8 | 16.1 | 225 | 0.015 | 4.218 | 0.064 | 2.52 | 0.03 | 1.28 | 0.06 | 3 |
| Tube-9 | 9 | 9.4 | 129 | 0.008 | 4.806 | 0.068 | 2.58 | 0.03 | 1.33 | 0.01 | 3 |
| Tube-10 | 10 | 7.9 | 66 | 0.005 |  |  |  |  |  |  |  |
| Tube-11 | 11 | 4.3 | 33 | 0.003 |  |  |  |  |  |  |  |
| Tube-12 | 12 | 3.7 | 17 | 0.002 |  |  |  |  |  |  |  |
| Tube-13 | 13 | 1.0 | 10 | 0.002 |  |  |  |  |  |  |  |
| Tube-14 | 14 | 3.1 | 6 | 0.002 |  |  |  |  |  |  |  |
| Tube-15 | 15 | 1.8 | 3 | 0.001 |  |  |  |  |  |  |  |
| Cell-final |  | 13.50 | 27 | 0.001 |  |  |  |  |  |  |  |
| Total Mg  Mass (∑) |  |  | 19706 |  |  |  |  |  |  |  |  |

* original Mg mass in diffusion cell was 18940 ug (9470ppm/ml, 2ml), the relative difference between the original Mg mass in diffusion cell and the total Mg mass in diffusion tubes after the diffusion experiment was 4.1%.

Table A3-5: Diffusion experiment for MgCl_2_ at 70°C, through membrane of MWCO=3500 towards deionized water.

| **Sample**  **No.** | **Time**  **(hours)** | **Mg conc.**  **(ppm)** | **Mg mass**  **(ug)** | **f** | **-lnf** | **lnf-err** | **d26Mg** | **2SD** | **d25Mg** | **2SD** | **n** |
| --- | --- | --- | --- | --- | --- | --- | --- | --- | --- | --- | --- |
|  | 0 |  |  |  |  |  | 2.23 | 0.05 | 1.16 | 0.06 |  |
| Tube-1 | 0.5 | 120.59 | 6029 | 0.676 | 0.391 | 0.069 | 2.07 | 0.04 | 1.06 | 0.06 | 4 |
| Tube-2 | 1 | 81.43 | 4072 | 0.456 | 0.785 | 0.069 | 2.19 | 0.06 | 1.13 | 0.02 | 4 |
| Tube-3 | 1.5 | 59.52 | 2976 | 0.296 | 1.219 | 0.070 | 2.20 | 0.04 | 1.14 | 0.03 | 4 |
| Tube-4 | 2 | 33.75 | 1688 | 0.205 | 1.587 | 0.068 | 2.27 | 0.05 | 1.17 | 0.04 | 4 |
| Tube-5 | 2.5 | 26.49 | 1325 | 0.133 | 2.017 | 0.067 | 2.41 | 0.01 | 1.22 | 0.04 | 4 |
| Tube-6 | 3 | 17.31 | 865 | 0.086 | 2.449 | 0.065 | 2.47 | 0.05 | 1.25 | 0.07 | 4 |
| Tube-7 | 3.5 | 11.88 | 594 | 0.054 | 2.913 | 0.063 | 2.51 | 0.05 | 1.30 | 0.04 | 4 |
| Tube-8 | 4 | 7.90 | 395 | 0.033 | 3.412 | 0.061 | 2.61 | 0.03 | 1.33 | 0.01 | 4 |
| Tube-9 | 4.5 | 5.73 | 286 | 0.018 | 4.045 | 0.060 | 2.62 | 0.03 | 1.34 | 0.03 | 4 |
| Tube-10 | 5 | 3.23 | 161 | 0.009 | 4.732 | 0.063 | 2.72 | 0.05 | 1.37 | 0.04 | 4 |
| Tube-11 | 5.5 | 1.44 | 72 | 0.005 | 5.313 | 0.065 | 2.77 | 0.01 | 1.40 | 0.02 | 4 |
| Tube-12 | 6 | 0.78 | 39 | 0.003 |  |  |  |  |  |  |  |
| Tube-13 | 6.5 | 0.38 | 19 | 0.002 |  |  |  |  |  |  |  |
| Tube-14 | 7 | 0.09 | 5 | 0.002 |  |  |  |  |  |  |  |
| Cell-final |  | 14.35 | 29 | 0.000 |  |  |  |  |  |  |  |
| Total Mg  Mass (∑) |  |  | 18525 |  |  |  |  |  |  |  |  |

* original Mg mass in diffusion cell was 18940 ug (9470ppm/ml, 2ml), the relative difference between the original Mg mass in diffusion cell and the total Mg mass in diffusion tubes after the diffusion experiment was 2.2%.

Table A3-6: Diffusion experiment for Mg(NO_3_)_2_ at 25°C, through membrane of MWCO=3500 towards deionized water.

| **Sample**  **No.** | **Time**  **(hours)** | **Mg conc.**  **(ppm)** | **Mg mass**  **(ug)** | **f** | **-lnf** | **lnf-err** | **d26Mg** | **2SD** | **d25Mg** | **2SD** | **n** |
| --- | --- | --- | --- | --- | --- | --- | --- | --- | --- | --- | --- |
|  | 0 |  |  |  |  |  | 0.00 | 0.07 | 0.00 | 0.06 | 4 |
| Tube-1 | 1.5 | 144.36 | 7218 | 0.619 | 0.47965 | 0.066 | -0.10 | 0.01 | -0.06 | 0.02 | 4 |
| Tube-2 | 3 | 86.30 | 4315 | 0.391 | 0.93905 | 0.066 | -0.01 | 0.06 | -0.01 | 0.04 | 4 |
| Tube-3 | 4.5 | 55.99 | 2799 | 0.243 | 1.41469 | 0.065 | 0.03 | 0.08 | 0.02 | 0.03 | 4 |
| Tube-4 | 6 | 35.26 | 1763 | 0.150 | 1.89712 | 0.065 | 0.13 | 0.08 | 0.08 | 0.05 | 4 |
| Tube-5 | 7.5 | 21.89 | 1095 | 0.092 | 2.38597 | 0.064 | 0.14 | 0.03 | 0.07 | 0.02 | 4 |
| Tube-6 | 9 | 14.38 | 719 | 0.054 | 2.91877 | 0.064 | 0.21 | 0.02 | 0.11 | 0.05 | 4 |
| Tube-7 | 10.5 | 8.37 | 419 | 0.032 | 3.44202 | 0.064 | 0.33 | 0.04 | 0.16 | 0.05 | 4 |
| Tube-8 | 12 | 4.96 | 248 | 0.019 |  |  |  |  |  |  |  |
| Tube-9 | 13.5 | 2.96 | 148 | 0.011 |  |  |  |  |  |  |  |
| Tube-10 | 15 | 1.63 | 81 | 0.007 |  |  |  |  |  |  |  |
| Tube-11 | 16.5 | 1.03 | 52 | 0.004 |  |  |  |  |  |  |  |
| Tube-12 | 18 | 0.60 | 30 | 0.003 |  |  |  |  |  |  |  |
| Tube-13 | 19.5 | 0.42 | 21 | 0.002 |  |  |  |  |  |  |  |
| Cell-final |  | 14.35 | 29 | 0.002 |  |  |  |  |  |  |  |

Table A3-7: Diffusion experiment for MgSO_4_ at 25°C, through membrane of MWCO=3500 towards deionized water.

| **Sample**  **No.** | **Time**  **(hours)** | **Mg conc.**  **(ppm)** | **Mg mass**  **(ug)** | **f** | **-lnf** | **lnf-err** | **d26Mg** | **2SD** | **d25Mg** | **2SD** | **n** |
| --- | --- | --- | --- | --- | --- | --- | --- | --- | --- | --- | --- |
|  | 0 |  |  |  |  |  | 2.29 | 0.07 | 1.14 | 0.02 | 3 |
| Tube-1 | 5 | 189.35 | 9468 | 0.619 | 0.480 | 0.071 | 2.13 | 0.01 | 1.06 | 0.00 | 3 |
| Tube-2 | 10 | 90.75 | 4537 | 0.434 | 0.836 | 0.070 | 2.20 | 0.01 | 1.13 | 0.06 | 3 |
| Tube-3 | 15 | 66.78 | 3339 | 0.297 | 1.213 | 0.069 | 2.24 | 0.04 | 1.15 | 0.07 | 3 |
| Tube-4 | 20 | 50.85 | 2543 | 0.194 | 1.641 | 0.070 | 2.24 | 0.05 | 1.18 | 0.01 | 3 |
| Tube-5 | 25 | 30.17 | 1508 | 0.132 | 2.024 | 0.069 | 2.34 | 0.04 | 1.21 | 0.04 | 3 |
| Tube-6 | 30 | 18.93 | 946 | 0.094 | 2.369 | 0.065 | 2.38 | 0.03 | 1.36 | 0.48 | 3 |
| Tube-7 | 35 | 17.83 | 892 | 0.057 | 2.861 | 0.065 | 2.39 | 0.02 | 1.22 | 0.01 | 3 |
| Tube-8 | 40 | 11.30 | 565 | 0.034 | 3.377 | 0.067 | 2.42 | 0.05 | 1.23 | 0.03 | 3 |
| Tube-9 | 45 | 6.76 | 338 | 0.020 | 3.894 | 0.072 | 2.49 | 0.05 | 1.26 | 0.08 | 3 |
| Tube-10 | 50 | 3.80 | 190 | 0.013 | 4.374 | 0.080 | 2.51 | 0.05 | 1.27 | 0.08 | 3 |
| Tube-11 | 55 | 2.28 | 114 | 0.008 | 4.833 | 0.098 | 2.51 | 0.05 | 1.26 | 0.01 | 3 |
| Tube-12 | 60 | 0.88 | 44 | 0.006 |  |  |  |  |  |  |  |
| Tube-13 | 65 | 0.48 | 24 | 0.005 |  |  |  |  |  |  |  |
| Tube-14 | 70 | 0.10 | 5 | 0.005 |  |  |  |  |  |  |  |
| Tube-15 | 75 | 0.08 | 4 | 0.005 |  |  |  |  |  |  |  |
| Tube-16 | 80 | 0.09 | 4 | 0.005 |  |  |  |  |  |  |  |
| Cell-final |  | 56.84 | 114 | 0.005 |  |  |  |  |  |  |  |
| Total Mg  Mass (∑) |  |  | 24635 |  |  |  |  |  |  |  |  |

* original Mg mass in diffusion cell was 24506ug (12253ppm/ml, 2ml), the relative difference between the original Mg mass in diffusion cell and the total Mg mass in diffusion tubes after the diffusion experiment was 0.5%.

Table A3-8: Diffusion experiment for MgCl_2_ at 25°C, through membrane of MWCO=3500 towards 0.8M HCl.

| **Sample**  **No.** | **Time**  **(hours)** | **Mg conc.**  **(ppm)** | **Mg mass**  **(ug)** | **f** | **-lnf** | **lnf-err** | **d26Mg** | **2SD** | **d25Mg** | **2SD** | **n** |
| --- | --- | --- | --- | --- | --- | --- | --- | --- | --- | --- | --- |
|  | 0 |  |  |  |  |  | 2.23 | 0.05 | 1.16 | 0.06 | 3 |
| Tube-1 | 3.5 | 210.09 | 7218 | 0.461 | 0.773 | 0.061 | 2.24 | 0.12 | 1.11 | 0.07 | 3 |
| Tube-2 | 7 | 86.05 | 4315 | 0.241 | 1.424 | 0.061 | 2.32 | 0.06 | 1.18 | 0.01 | 3 |
| Tube-3 | 10.5 | 43.78 | 2799 | 0.129 | 2.051 | 0.060 | 2.42 | 0.04 | 1.21 | 0.04 | 3 |
| Tube-4 | 14 | 23.35 | 1763 | 0.069 | 2.677 | 0.059 | 2.46 | 0.04 | 1.25 | 0.04 | 3 |
| Tube-5 | 17.5 | 13.44 | 1095 | 0.034 | 3.372 | 0.059 | 2.54 | 0.01 | 1.28 | 0.01 | 3 |
| Tube-6 | 21 | 6.85 | 719 | 0.017 | 4.090 | 0.058 | 2.68 | 0.03 | 1.33 | 0.06 | 3 |
| Tube-7 | 24.5 | 3.54 | 419 | 0.008 | 4.870 | 0.058 | 2.77 | 0.07 | 1.39 | 0.04 | 3 |
| Tube-8 | 28 | 1.65 | 248 | 0.003 | 5.672 | 0.058 | 2.80 | 0.04 | 1.41 | 0.03 | 3 |
| Tube-9 | 31.5 | 0.75 | 148 | 0.002 | 6.483 | 0.059 | 2.94 | 0.06 | 1.46 | 0.03 | 3 |
| Tube-10 | 35 | 0.32 | 81 | 0.001 | 7.240 | 0.060 | 3.03 | 0.03 | 1.51 | 0.04 | 3 |
| Tube-11 | 38.5 | 0.13 | 52 | 0.000 |  |  |  |  |  |  |  |
| Tube-12 | 42 | 0.06 | 30 | 0.000 |  |  |  |  |  |  |  |
| Cell-final |  | 2.09 | 29 | 0.002 |  |  |  |  |  |  |  |
| Total Mg  Mass (∑) |  |  | 19501 |  |  |  |  |  |  |  |  |

* original Mg mass in diffusion cell was 18940 ug (9470ppm/ml, 2ml), the relative difference between the original Mg mass in diffusion cell and the total Mg mass in diffusion tubes after the diffusion experiment was 3.0%.
