# Supplementary material for: Kinetic fractionation of Mg isotopes during chemical diffusion in aqueous solutions: A reappraisal
Source: Fundam Res. 2024 Dec 25;6(4):2387–97. doi: 10.1016/j.fmre.2024.11.010 (PMC13424388; doi:10.1016/j.fmre.2024.11.010)
Supplement: Supplementary file 3 [file mmc3.docx]

**Appendix A4**. Data for adsorption-desorption experiments of 40ppm Mg solutions onto the membranes of the two different diffusion cells

|  | **Mass (μg)** | **δ^26^Mg** | **2SD** | **δ^25^Mg** | **2SD** |
| --- | --- | --- | --- | --- | --- |
|  |  |  |  |  |  |
| **Experiment of MgSO_4_ adsorption** | | | | | |
| MgSO_4_ original solution |  | 2.34 | 0.06 | 1.19 | 0.05 |
| Mg adsorbed on membrane of MWCO =3500 | **2.28** | 2.15 | 0.19 | 1.11 | 0.14 |
| Mg adsorbed on membrane of MWCO =20000 | **1.97** | 2.27 | 0.09 | 1.17 | 0.09 |
|  |  |  |  |  |  |
| **Experiment of MgCl_2_ adsorption** | | | | | |
| MgCl_2_ original solution |  | 1.12 | 0.07 | 0.57 | 0.02 |
| Mg adsorbed on membrane of MWCO =3500 | **3.24** | 0.98 | 0.03 | 0.51 | 0.03 |
| Mg adsorbed on membrane of MWCO =20000 | **3.82** | 1.02 | 0.28 | 0.51 | 0.24 |
|  |  |  |  |  |  |
| **Experiment of Mg(NO_3_)_2_ adsorption** | | | | | |
| Mg(NO_3_)_2_ original solution |  | -0.01 | 0.07 | 0.01 | 0.03 |
| Mg adsorbed on membrane of MWCO =3500 | **3.46** | -0.03 | 0.15 | -0.03 | 0.15 |
| Mg adsorbed on membrane of MWCO =20000 | **3.75** | -0.04 | 0.14 | -0.05 | 0.10 |
